# Border malaria in China: knowledge and use of personal protection by minority populations and implications for malaria control: a questionnaire-based survey

**DOI:** 10.1186/1471-2458-8-344

**Published:** 2008-10-01

**Authors:** Sarah J Moore, Xia Min, Nigel Hill, Caroline Jones, Zhang Zaixing, Mary M Cameron

**Affiliations:** 1 DCVBU, London School of Hygiene and Tropical Medicine, London, UK; 2 Public Health Entomology, Ifakara Health Institute, Ifakara, Tanzania; 3 School of Biological and Biomedical Sciences, Durham University, Durham, UK; 4 Simao Institute of Parasitic Disease Control, Simao, PR China

## Abstract

**Background:**

Malaria control in remote, forested areas of the Mekong region relies on personal protection from mosquito bites. Uptake of these methods may be limited by knowledge of the link between mosquitoes and malaria as well as social and economic aspects. Understanding barriers to uptake will inform malaria control programmes on targets for improvement of delivery.

**Methods:**

A total 748 key respondents: health providers and village heads, from 187 villages and 25 different ethnic groups, were interviewed using structured questionnaires. Differences in use of personal protection, and knowledge of malaria between groups were analysed using chi-square; and binary logistic regression used for multivariate analysis.

**Results:**

Malaria knowledge was poor with 19.4% of women and 37.5% of men linking mosquitoes with malaria, although 95.6% knew one or more methods of mosquito control. Virtually all respondents used personal protection at some time during the year; and understanding of malaria transmission was strongly associated with bednet use. Those working in forest agriculture were significantly more likely to know that mosquitoes transmit malaria but this did not translate into a significantly greater likelihood of using bednets. Furthermore, use of personal protection while woing outdoors was rare, and less than 3% of respondents knew about the insecticide impregnation of bednets. The use of bednets, synthetic repellents and mosquito coils varied between ethnic groups, but was significantly more frequent among those with higher income, more years of education and permanent housing. The reported use of repellents and coils was also more common among women despite their low knowledge of malaria transmission, and low likelihood of having heard information on malaria within the last year.

**Conclusion:**

The use of personal protection must be increased, particularly among outdoor workers that have higher malaria risk. However, personal protection is widely used and widely accepted to prevent nuisance biting mosquitoes, with the major barrier to use being affordability. Therefore, social marketing campaigns aimed at women and those that work outdoors that provide highly subsidised products, especially insecticide impregnation kits for bednets and hammock nets are most likely to succeed in lowering malaria morbidity among non Han-Chinese groups in rural China.

## Background

Despite the very significant regional decline in reported malaria cases and deaths due to Malaria in the Greater Mekong sub-region during the 1990s, the disease remains an important public health problem in six countries of the Region [1]. In the six endemic Asian countries, Cambodia, China (Yunnan Province), the Lao People's Democratic Republic, Malaysia, the Philippines and Vietnam, most malaria cases occur in remote forested and hilly areas where mosquito vectors are abundant and health services are frequently inadequate. It is estimated that 36% of the population of the Greater Mekong Sub region reside in these areas without adequate vector control [2]. Malaria prevalence is highest among ethnic minorities, migrants and forest workers, with the most vulnerable members of the population being pregnant women, the very poor and the malnourished [3].

The current study investigates knowledge of malaria prevention and use of personal protection measure among 25, ethnic minority populations in Yunnan Province in Southwest China. Most Chinese are Han-Chinese, but the populations interviewed during this study are recognised as ethnic nationalities by the state: having culture, language and lifestyle unique to their groups [4]. As the groups are autonomous, one group may reside in several countries. They frequently cross international borders to visit family, for cross border marriages, and to conduct trade [4], where they often contract malaria while travelling and import it to their villages [5]. Yunnan Province is one of the two remaining areas of China with high annual transmission of both *P. vivax *and *P. falciparum*; the other being Hainan Island. In 2004, the annual reported malaria incidence was 3.09/10,000 [6], although the estimated number of actual cases is at least 18 times greater [7]. Malaria is a particularly severe social and health problem along the border with Myanmar, where mobile workers move back and forth across the border and malaria control is weak. One-third of malaria cases in China came from Yunnan province in 2005, and about a quarter of these were actually infected in Myanmar during trips to visit relatives and conduct business [8].

The mobile populations within the border areas are often vulnerable and of low economic status. However, control of disease and effective treatment is problematic since it is difficult to locate, diagnose and treat infected people in these populations [9]. Annual GDP per capita of the rural population in the border region is one of the lowest in China at <US$100 [10]. Over half of per capita health expenditure is provided by out-of-pocket contributions [11]. Furthermore, 39% of people in rural-poor areas do not seek health care because of economic difficulties [12], and it is estimated that each bout of malaria severely reduces loss of earnings; with a 1.45% loss of annual income per episode experienced [13]. Thus, the prevention of malaria morbidity among minority and mobile populations is a priority for malaria control programmes in this region.

Malaria control is complicated by the fact that the local malaria vector mosquitoes: *Anopheles. dirus *A, *An. minimus *A and *An. minimus *C [14], exhibit behaviours that limit their control through traditional methods such as indoor residual spraying (IRS). These vectors breed in scattered forest sites [15], are exophilic and exophagic [16]; and their tendency to feed in the early evening reduces the impact of bednets [16,17]. Targeted personal protection may have an important role in preventing malaria that is transmitted by exophilic and exophagic vectors that bite early in the evening when people are still outdoors [18,19].

Yunnan is part of the Mekong Roll Back Malaria (RBM) initiative that highlighted the need to evaluate innovative treatments to protect those at risk. RBM began in 1999, and the vector control component focuses on improving information about, access to, and use of insecticide treated bednets because bednets have been used for many years in Yunnan without insecticide [20]. The initiative aims to develop better systems for collecting relevant information to make appropriate decisions on vector control and personal protection, so that the limited resources are targeted at those most in need [21]. Therefore, the aim of the current study is to investigate the existing use of, motivation for using and perceptions of personal protection among rural ethnic-minority populations; in order to identify key areas where uptake of personal protection can be maximised through dissemination of knowledge or improvement of provision.

## Methods

### Study Design

A survey was performed between May and August 2003, in those counties with highest malaria incidence. For each of the twenty five minority populations, approximately ten villages were visited. However, for some groups it was not possible to visit all ten villages due to road conditions and analysis was weighted accordingly. The villages were chosen at random from a list, and interviews were conducted with four key people in each of the 187 villages visited, with 748 interviews conducted in total. The respondents selected were the head of the village and individuals suggested by village heads because they were herbalists or healers. These were selected as respondents because they are most likely to have information on health issues in the region, and because they give information about diseases to their communities, it is important to know what knowledge and practices they have. The survey was designed to rapidly gather information from a large cross section of the population and so those people who are most likely to disseminate information within a community were selected. The survey was designed to measure associations between socioeconomic factors, knowledge and practice. The survey was conducted during the rains because both mosquito numbers and malaria incidence is highest at this time [22], thus the subject matter would be fresh in peoples' minds.

### Data collection

Data on the household environment, knowledge and perceptions of personal protection and risk factors for malaria was collected using a structured interview with data entered into questionnaires. This method was favoured over focus groups for health research in this region because replies to questions may sometimes be biased to include what the respondent perceives is a socially desirable response [23]. Reference numbers were used to ensure participant confidentiality.

Structured observations should only be undertaken following exploratory research such as a pilot survey [24], but his was beyond the scope of the study due to time and money constraints. Therefore, the expertise of individuals who had performed this kind of survey previously in the area was resourced. Interviews were conducted by anthropologists from Simao Institute of Parasitic Disease Control who have worked extensively among minority populations in this region and that speak several local dialects. Participants were asked about methods they used to prevent mosquito bites, and their knowledge of malaria. Knowledge of malaria was measured by asking questions about mosquito breeding sites, how malaria is transmitted and preventative measures. Answers were noted using checkboxes but questions were posed in an open fashion to prevent bias of answers. The questionnaire was translated into Mandarin Chinese, and then the questions were translated on the day into local dialect using a translator when necessary, although most respondents spoke Mandarin. The questionnaires were coded using Arabic numbers and all data were entered into a data base using coding to ensure blind data entry. Any sections where informants supplied additional information were back-translated into English and entered separately. Data were entered using Epi Info 2002 and analysis was performed using SPSS 13.0. Pairs of variables were analysed using chi square to test for significant relationships, followed by stepwise multivariate binary logistic regression of significant variables adjusted for altitude (above and below 1500 m), ethnic group and village.

### Ethical Considerations

Ethical clearance for the study has been provided by Yunnan Research Ethical Committee and the LSHTM Research Ethics Committee. Prior to the study, each potential interviewee had a full explanation regarding the reason for the study, procedure and time required to perform the interview, and was given the opportunity to opt out. Identification numbers were used to protect the participants' identities and make data analysis simpler.

## Results

The majority of the respondents were male (62.7%), working in agriculture (73.5%), with an annual household income greater than 1200Y (86.9%). Most families comprised 3–5 individuals, who had been living in the area for more than 10 years (93.1%), and the most common housing type was the traditional open two storey housing found in Yunnan, which is constructed of wood and allows mosquitoes ready access (listed as "permanent open" in Table 1). The majority of villages were surrounded by crops (76%), rice fields (87%) and forest (63.8%); therefore they are in close proximity to mosquito breeding sites. Educational level was low: 30% of respondents had received no schooling, with 38% receiving primary education and 27% attending secondary education. There was significant gender disparity in education: 47% of females receiving no education versus 22% of males (Fishers exact test *P *< 0.0001). Educational level was significantly higher in the youngest age group, with more young people having received both primary and secondary education than those in older age groups (Fishers exact test *P *< 0.0001) (Table 1).

**Table 1:** Binary logistic regression to identify key determinants of malaria knowledge and personal protection use

**Variable**	**n (%)**^**1**^	**Know ** **mosquito** **transmits** **malaria^2^**	**Odds ** **ratio**	**Recently ** **heard** **info on** **malaria^3^**	**Odds ** **ratio**	**Uses ** **bednets^4^**	**Odds ** **ratio**	**Uses ** **coils^5^**	**Odds ** **ratio**	**Uses ** **repellents^6^**	**Odds ** **ratio**
**Gender**			p = 0.001		p = 0.003		p = 0.092		p < 0.001		p < 0.0001
Male	480 (62.7)	180 (37.5)	1	085 (17.7)	1	313 (65.3)	1	234 (48.8)	1	232 (48.3)	1
Female	268 (35.0)	052 (19.4)	0.443 (0.279, 0.703)	017 (06.3)	0.352 (0.176, 0.706)	185 (69.0)	1.457 (0.938, 2.321)	160 (59.7)	2.331(1.500, 3.624)	172 (64.2)	2.578 (1.664, 3.994)

**Age**			p = 0.978		p = 0.342		p = 0.711		p = 0.143		p = 0.066
< 30	119 (14.4)	030 (27.3)	1	011 (10.0)	1	078 (70.9)	1	048 (43.6)	1	048 (43.6)	1
30 to 50	378 (49.4)	116 (30.7)	1.025 (0.650, 1.684)	062 (16.4)	0.628 (0.330, 1.195)	260 (68.8)	0.858 (0.546, 0.346)	205 (54.2)	1.332 (0.891, 1.991)	202 (53.4)	1.574 (1.002, 2.471)
> 50	255 (33.3)	084 (32.9)	1.072 (0.529, 2.174)	029 (11.4)	0.830 (0.329, 2.095)	158 (62.0)	0.795 (0.432, 1.464)	138 (54.1)	1.808 (0.952, 3.431)	150 (58.8)	2.022 (1.002, 2.471)

**Income**			p = 0.656		p = 0.127		p = 0.004		p < 0.0001		p < 0.0001
< 600 Y	013 (01.7)	002 (15.4)	1	003 (23.1)	1	003 (23.1)	1	001 (07.7)	1	001 (07.7)	1
600–1200 Y	056 (07.3)	010 (17.9)	1.313 (0.619, 2.785)	003 (05.4)	2.864 (0.953, 8.613)	024 (42.9)	1.774 (0.911, 03.454)	010 (17.9)	04.257 (1.838, 009.857)	015 (26.8)	02.849 (1.347, 006.024)
Over 1200 Y	665 (86.9)	216 (32.5)	1.858 (0.415, 8.320)	094 (14.1)	0.374 (0.065, 2.138)	461 (69.3)	8.012 (1.784, 35.977)	380 (57.1)	21.584 (2.373, 196.316)	385 (57.9)	27.993 (3.212, 243.985)

**Housing**			p = 0.629		p = 0.015		p = 0.386		p = 0.0001		p = 0.012
Temporary	059 (07.7)	009 (15.3)	1	009 (15.3)	1	033 (55.9)	1	013 (22.0)	1	019 (32.2)	1
Semi-perm	057 (07.5)	015 (26.3)	1.347 (0.645, 2.813)	010 (17.5)	1.049 (0.364, 03.020)	029 (50.9)	0.818 (0.393, 1.702)	014 (24.6)	1.586 (0.789, 03.186)	015 (26.3)	1.654 (0.891, 03.069)
Perm Open	539 (70.5)	166 (30.8)	1.617 (0.611, 4.279)	058 (10.8)	2.852 (1.360, 05.983)	383 (71.1)	1.418 (0.511, 3.934)	305 (56.6)	4.847 (1.769, 13.283)	308 (57.1)	4.955 (1.765, 13.916)
Perm Closed	088 (11.5)	040 (45.5)	2.240 (0.575, 8.742)	024 (27.3)	2.476 (0.607, 10.094)	050 (56.8)	1.215 (0.481, 3.070)	058 (65.9)	5.895 (2.002, 17.361)	057 (64.8)	3.222 (1.14, 09.075)

**Education**			p = 0.001		p = 0.002		p = 0.01		p < 0.0001		p = 0.004
None	232 (30.3)	043 (18.5)	1	015 (06.5)	1	139 (59.9)	1	096 (41.4)	1	114 (49.1)	1
Primary	291 (38.0)	088 (30.2)	1.548 (0.944, 2.536)	040 (13.7)	2.020 (1.122, 3.637)	194 (66.7)	2.063 (1.218, 3.493)	149 (51.2)	2.171 (1.311, 3.594)	057 (54.0)	1.241 (0.751, 2.049)
Secondary	209 (27.3)	091 (43.5)	2.980 (1.666, 5.332)	045 (21.5)	3.412 (1.677, 6.942)	160 (76.6)	2.496 (1.352, 4.610)	142 (67.9)	4.216 (2.445, 7.268)	128 (61.2)	2.465 (1.437, 4.229)

**Occupation**			p = 0.001		Not calculated		p = 0.168		p = 0.1		p = 0.001
Agriculture	562 (73.5)	157 (28.9)	1	071 (13.1)		369 (68.0)	1	284 (51.1)	1	300 (54.0)	1
Forestry	031 (04.0)	019 (61.3)	2.194 (0.373,12.909)	008 (25.8)		017 (63.0)	2.196 (0.255, 018.925)	011 (40.7)	0.374 (0.041, 3.441)	006 (19.4)	0.210 (0.038, 01.150)
Plantation	027 (03.5)	005 (18.5)	0.131 (0.030, 00.567)	008 (29.6)		023 (74.2)	4.009 (0.101, 158.693)	009 (29.0)	0.348 (0.040, 3.056)	005 (18.5)	0.680 (0.019, 23.960)
Labourer	014 (01.8)	003 (25.0)	0.722 (0.210, 02.475)	000 (00.0)		009 (75.0)	4.051 (0.694, 023.657)	004 (28.6)	0.152 (0.023, 1.015)	005 (35.7)	0.295 (0.054, 01.621)
Other	096 (12.5)	044 (37.9)	0.645 (0.173, 02.407)	013 (11.0)		066 (56.9)	2.206 (0.362, 013.452)	075 (74.3)	0.113 (0.017, 0.767)	077 (76.2)	1.746 (0.230, 13.242)

**Altitude**			P < 0.0001		p < 0.0001		P < 0.0001		p = 0.183		p < 0.0001
< 1200 m	314 (42.0)	134 (42.7)	1	060 (19.3)	1	238 (75.8)	1	172 (54.8)	1	143 (45.5)	1
> 1200 m	424 (58.0)	098 (22.6)	0.392 (0.285, 0.538)	042 (09.7)	0.449 (0.294, 0.687)	260 (59.9)	0.447 (0.346, 0.658)	222 (51.2)	0.865 (0.646, 1.157)	261 (60.1)	1.804 (1.345, 2.420)

The number and percentage of respondents in each socioeconomic category was stratified by gender and age of respondent, housing quality, annual household income, occupation of the head of the household, educational level of respondent, occupation of the head of household and the village altitude (> or < 1500 m). The likelihood of those respondents in each category correctly identifying night-biting mosquitoes as the cause of malaria^1^; having heard information on malaria in the last year^2^; and answering yes to the question do you use bednets^3^, coils^4^, and repellents^5 ^to protect yourself against mosquitoes were calculated with binary logistic regression.

Only 31% of respondents knew that mosquitoes transmit malaria, and knowledge was significantly lower among women (O.R. 0.443, 95% C.I.: 0.279–0.703, *P *= 0.001), who were also less likely to have heard information on malaria within the last 12 months (O.R. 0.352, 95% C.I.: 0.176–0.706, *P *= 0.003) (Table 1). There was also a strong association between recalling hearing information on malaria and knowing that mosquitoes transmit malaria (O.R. 7.414, 95% C.I.: 4.669–11.442, *P *< 0.0001).

Almost all of the households perceived mosquitoes as a nuisance (97.1%) and women were more aware of this than men (O.R. 6.104, 95% C.I.: 1.185–31.436, *P *= 0.031), but only 21.9% of respondents mentioned that mosquitoes are a problem because they transmit malaria. Those who lived close to rice fields were also more likely to perceive mosquitoes as a nuisance (O.R. = 5.085, 95% C.I.: 2.111–12.246, *P *= 0.001), but were less likely to perceive mosquitoes as a problem because they cause malaria (O.R. 0.480, 95% C.I.: 0.302–0.762, P = 0.002). Those who understood that malaria was transmitted by mosquitoes were more likely to use bednets (OR 1.572, 95% C.I.: 1.117–2.212, *P *= 0.005), but not repellents or mosquito coils. Use of all three methods of personal protection were strongly related to receiving primary and secondary education, and even more strongly predictive of use was higher income (Table 1).

Although knowledge that mosquitoes transmit malaria was low, knowledge of mosquito control methods was high. When asked (without prompting), "What methods do you know that can be used to prevent mosquito nuisance?" 80% of respondents mentioned bednets, 75.5% mentioned indoor residual spraying (IRS) and 65% mentioned repellents as methods for controlling mosquitoes. However, when this question was rephrased later in the questionnaire as "how can you protect yourself against malaria" 43% replied that they didn't know, 12.5% answered IRS, 8.6% mentioned bednets and 19% mentioned drugs such as chloroquine.

**Figure 1:**
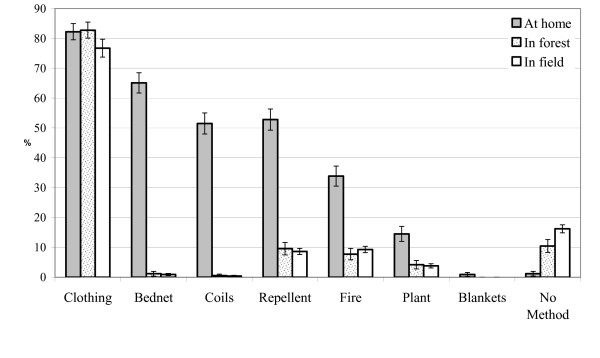
**Differences in the use of personal protection when at home, or outdoors in the forest or field where malaria risk is higher**. The figure represents the percentage of respondents that use personal protection when at home or outdoors. Bars denote the mean and 95% c.i. percentage of responses (n= 748). The respondents were asked "Do you try to protect yourself from mosquito bites? If you do, how do you protect yourself when you are at home? How do you try to protect yourself when you enter the forest? How do you protect yourself when you are working in the fields? Respondents could make multiple responses.

Although most people used personal protection, this was largely confined to use inside of houses, and use of personal protection outdoors besides wearing long clothing was rare (Figure 1). Importantly, those working in occupations where they are likely to encounter mosquitoes used insect repellents less frequently than those in agriculture i.e. foresters (OR 0.210, 95% C.I.: 0.038–1.150, *P *= 0.001) and plantation workers (OR 0.68, 95% C.I.: 0.019–23.960, *P *= 0.001) (Table 1). This result was surprising, since foresters had the best knowledge of malaria transmission: 61.3% identified mosquitoes as the cause of malaria compared to 37.9% who were employed in 'other' indoor occupations including medicine and business (Table 1), and more than twice as many people engaged in forestry or plantation agriculture had heard information on malaria when compared to other occupations (Pearson Chi Square *P *= 0.017). Importantly, those working in forestry and plantation agriculture were four times more likely to use bednets, although the association was not significant (Table 1).

Bednet use varied greatly between different groups, ranging from 3.6% among the Zang to 100% among the Shui; with a mean of 66.5% of respondents using bednets at home; and 97.4% of users said that they used the nets because they were effective rather than cheap. Of those who used nets, 62.7% used them to protect themselves from mosquitoes with low numbers using them for privacy (1.8%), to stop dust (7.6%) and for warmth (4.8%). The same trend was observed among repellent and coil users with 95.8% and 96.2%, respectively, of those respondents that used repellents or coils, perceiving them as effective. Almost all of the bednets used were unimpregnated because only 0.9% of households heads interviewed used a treated net, and only 3.1% knew about the insecticide treatment of bednets.

## Discussion and conclusion

Key informants were chosen for this survey because they are the information givers on health issues in remote areas, and traditional healers were selected as they may also treat those with malaria. It is of particular importance to identify the knowledge of malaria held by these groups as they disseminate knowledge to others in the community. However, it is appreciated that the survey may not be extrapolated to the whole autonomous population of Yunnan, because the interviewees are not representative of the population as a whole, with males and older people receiving over representation.

The two most important predictors of use of personal protection were income and education. Overall more educated people used personal protection more frequently and also had better knowledge of malaria. A similar trend was found by a survey conducted in Laos which showed that education was predictive of knowledge of transmission and use of bednets [25]. As there was no statistical relationship between education and income, it would appear that some health education is received whilst at school because those with more years of education were more likely to state mosquitoes as a cause of malaria. Disseminating malaria knowledge through schools is one of the areas of focus of the Mekong RBM project [20], and appears to be an effective strategy to improving community knowledge of malaria transmission. Additionally, those of higher educational status were more likely to have heard something about malaria in the last year. This indicates that there may also be language or literacy barriers resulting in low knowledge of malaria. Such barriers to knowledge may be overcome by the use of spoken messages about malaria prevention such as community plays and workshops [26].

Knowledge of malaria transmission was low overall, but women knew significantly less about the link between mosquitoes and malaria. However, this did not reduce their use of personal protection, a phenomenon that was also identified in a survey in Thailand [27]. The perception that mosquitoes are a nuisance may explain why women were more likely to use bednets, coils and repellents even though they had lower understanding of malaria transmission.

Several authors have recommended that malaria control for the region should target personal at men of working age, who have greater occupational exposure to mosquitoes [28-30]. Although it is likely that working men do bear a high malaria burden in the Mekong Region, there is evidence from some areas suggesting that mosquito exposure is similar among men and women, although the timing and location of their exposure might be different [31,32]. Women engage in many agricultural tasks alongside men – they provide 46.6% of agricultural labour in Yunnan, and also participate in forestry [33]. Since women are responsible for maintaining the household as well as performing agricultural tasks they tend to get up earlier than men, which also exposes them to early morning vector biting [34]. Some of the bias towards malaria burden in men may be due to underreporting in passive surveillance [35]. Women are less likely to seek health care due to a lack of recognition of women's health problems and barriers to mobility [34]; and are at high risk of malaria morbidity when pregnant [36-38]. Therefore targeting of personal protection information at both men and women may be more appropriate in this scenario. Although women generally have lower status than men in Yunnan, they are responsible for making household purchases [39,40]. This survey shows that they are most open to using personal protection methods. Improving women's knowledge and exposure to personal protection is important as they may be able to encourage use by other members of the family through their purchases. Again, the lower educational status of women suggests that verbal campaigns rather than written literature may be the best way to spread messages about malaria prevention and treatment.

It is interesting that those most exposed to mosquitoes i.e. those engaged in plantation agriculture and forestry only protect themselves from bites with long clothing whilst working, even though they had better knowledge of malaria transmission than those engaged in other occupations. However, they did use bednets more frequently (and they did not receive a higher income than other groups) which illustrates the importance of health education programmes in translating knowledge into practice. This lack of personal protection use away from home is common in Southeast Asia [30,34], and this phenomenon has been targeted as part of the RBM initiative, with NGOs investigating the potential for Social Marketing of hammock nets and deet (di-ethyl toulamide) based repellents for migratory populations [35]. Additionally, the mandatory use of personal protection in the field by plantation workers or those working for commercial logging companies in Southeast Asia could be negotiated as has occurred in Bolivia [41].

Health policy for the region targets increased coverage of long-lasting insecticidal nets [21], and the research clearly demonstrates a lack of knowledge about insecticide treatment of bednets among the surveyed population. Personal protection use among the participants of the survey is high despite low understanding of malaria transmission or control. This has been shown by several other surveys conducted among ethnic minority groups in Laos, Thailand and Myanmar [42-45]. There was a statistically significant relationship between mentioning that mosquitoes cause malaria and use of bednets, as was also seen in a similar survey conducted in Hainan Province [46]. However, the use of other methods of personal protection was motivated only by nuisance biting. The lack of relation between knowledge and practise, where nuisance is the greatest motivator for using personal protection, is widely recorded in the literature and has lead some researchers to advocate the use of protection measures independent of people's knowledge, stressing the importance of low price of interventions to facilitate purchase [47]. In fact, cost may be the most important hindrance to uptake of personal protection in this region as the strongest statistical association between factors was seen between income and personal protection use.

Health policy for the region targets increased coverage of long-lasting insecticidal nets [21], and the research clearly demonstrates a lack of knowledge about insecticide treatment of bednets among the surveyed population. The survey has shown that the use of insecticide impregnated nets among autonomous groups in Yunnan needs to increase alongside outdoor use of personal protection, especially among high risk groups such as forest workers. In an area like Yunnan where language and literacy can be a barrier, health education based on verbal communication such as village meetings and radio broadcasts for both men and women that coincide with bednet distribution may be required. However, personal protection is widely used and widely accepted, with the major barrier to use being affordability. Therefore social marketing campaigns with highly subsidised products are most likely to succeed.

## Competing interests

The authors declare that they have no competing interests.

## Authors' contributions

SJM designed the questionnaire, inputted and analysed data and wrote the manuscript. XM led the field collection team, assisted with questionnaire design and inputted data. NH conceived the study, assisted with study design, obtained ethic approval, liaised between institutes and revised the manuscript and obtained ethics approval. CJ assisted on design of questionnaire and revision of manuscript. ZZ designed the questionnaire and revised the manuscript. MCC designed the study and questionnaires and edited manuscript.

## Pre-publication history

The pre-publication history for this paper can be accessed here:


